# Update in the Diagnosis and Management of Ocular Surface Squamous Neoplasia (OSSN)

**DOI:** 10.3390/jcm14051699

**Published:** 2025-03-03

**Authors:** Michael Tsatsos, Chryseis Delimitrou, Ioannis Tsinopoulos, Nikolaos Ziakas

**Affiliations:** 2nd Ophthalmology Department, Aristotle University of Thessaloniki, Papageorgiou Hospital, Ag Pavlou 76, 564 29 Thessaloniki, Greece

**Keywords:** ocular surface squamous cell neoplasia, methods of management, 5-fluorouracil, mitomycin-C, interferon a2b

## Abstract

Ocular surface squamous neoplasia (OSSN) includes a variety of ocular surface tumors ranging from mild epithelial dysplasia to invasive squamous cell carcinoma. OSSN is one of the most frequent non-pigmented malignancies of the ocular surface. Debate persists between surgical excision and medical management concerning the optimal regimen for OSSN treatment, with surgical excision continuing to be the recognized standard of care in contemporary medicine. Medical and conservative therapy techniques have advanced significantly in recent years, leading to widespread use in everyday ophthalmology practice. This study aims to look into the efficacy of current treatment options for conjunctival squamous cell carcinoma and to evaluate the available evidence for the most up-to-date approach for the management of the disease.

## 1. Introduction

Lee et al. first introduced the term ocular surface squamous neoplasia (OSSN) in 1995 as a term used to include a plethora of ocular surface non-compound tumors [1,2]. Despite being relatively rare in the general population, OSSN is proven to be one of the most common non-pigmented malignancies of the ocular surface affecting the cornea, the conjunctiva, and the limbus [1,2,3,4]. The histological features may vary from mild dysplastic lesions, intraepithelial neoplasia, also known as carcinoma in situ, to squamous cell carcinoma [1,2,3]. Squamous cell carcinoma (SCC) is the most advanced type of OSSN and is considered common in certain areas, such as Australia and Africa [5]. The main risk factors for OSSN predominantly involve exposure to sunlight, outdoor professions, male gender, older age (with a majority of cases occurring in individuals over 60 years of age), smoking, immunosuppression, injury to the ocular surface, genetic predisposition, exposure to chemicals, and insufficiency of vitamin A [1,2,3,4,5]. The disease is strongly associated with the human papillomavirus (HPV) serotypes 6/11, 16, and 18 [6], but there is still some ambiguity on whether that may be accurate [7]. Immunosuppressed patients with HIV or AIDS appear to have higher occurrence, which explains the higher morbidity rates in younger women in Africa [2,8].

Conjunctival tumors are often categorized as congenital, acquired, malignant, pre-malignant, or benign [1,2,3,4,5]. The acquired lesions are typically classified into more specific categories based on the prevailing cell type. The most common kind of acquired lesions are epithelial tumors, with the majority being squamous cell tumors. Other prevalent types include melanocytic, vascular, and lymphoid tumors [2,4,5]. Shields et al. conducted a study at a single ocular oncology center and discovered that out of 771 non-melanocytic conjunctival tumors, OSSN was the most common non-pigmented tumor of the ocular surface, accounting for 23% (179 tumors) of the cases [4]. Conjunctival squamous cell carcinoma (SCC) is believed to arise from limbal stem cells [5,9] and is part of a larger group of ocular surface squamous neoplasias (OSSNs). It is one of the most common non-melanocytic tumors, along with lymphoma, and exhibits distinct clinical features [4,9].

**Figure 1:**
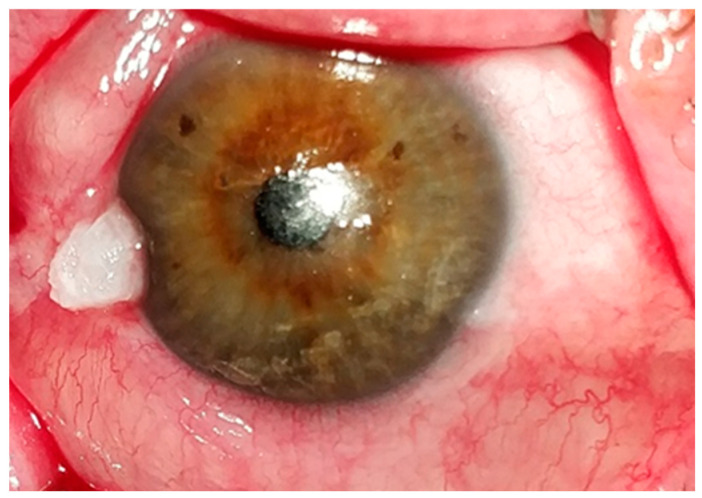
Leukoplakic type of OSSN.

A unilateral vascularized lesion is the most common clinical presentation, with bilateral or multifocal presentations occurring less frequently [2,4]. Lesion morphology may vary from gelatinous, leukoplakic, papillary, or nodular to nodular-ulcerative [2]. These lesions are typically located in the interpalpebral fissure medially or laterally and can be planar or textured, localized, or diffused (Figure 1) [2,4]. OSSN lesions mostly involve the cornea and/or bulbar conjunctiva and, less frequently, the tarsal conjunctiva [8]. In Caucasians, the mass is yellow-pink in color or darkly pigmented in those with dark complexions [2,4]. Abnormal tortuous dilated feeder arteries on the tumor could indicate malignant growth [2,9]. Occasionally, the tumor may penetrate the neighboring corneal epithelium, causing a foamy appearance [9,10]. Despite their rarity, nodular-ulcerative lesions are aggressive forms that strongly signal invasive neoplasia compared to other morphologies [2]. Furthermore, nodular and papillomatous lesions are frequently associated with more aggressive histopathologic grades [11]. Male gender, temporal location, and multifocality are tumor characteristics that contribute to higher levels of malignancy (i.e., enhanced mitotic activity, low cellular differentiation, and dysplasia) [10]. In general, elevated lesions indicate a higher degree of malignancy than flat lesions [11]. Symptoms of conjunctival SCC are typically non-specific, including redness, ocular discomfort, and visual impairment if the visual axis is involved [12].

The epidemiology of SCC varies geographically due to the varied causative agents affecting morbidity, which ranges from 0.3 to 35 per million people [13]. The incidence ranges from 0.02 per 100,000 in high-latitude locations to 3.5 per 100,000 at low latitudes near the Equator [14]. The reported global age-standardized rate for OSSN is 0.26 incidences per 100,000 per year with an upward trend over time. Africa has the highest incidence of OSSN globally (3.4 per 100,000/year), with males and females being equally affected, as opposed to other continents where the male disease predominates [15]. The increased risk in African women is strongly linked to a higher frequency of HIV and HPV infections [3,16,17]. Another study, conducted in Canada, found that the incidence of malignant OSSN is gradually increasing, likely due to an aging population [15].

SCC diagnosis can be challenging, particularly when it coexists with other ocular surface lesions, such as pinguecula and pterygia [18]. The gold standard for diagnosing OSSN is incisional or excisional biopsy with histological examination [10]. Other less invasive modalities include impression or exfoliative cytology [19,20], in vivo confocal microscopy (IVCM) [21], and high-resolution or ultra-high-resolution anterior segment optical coherence tomography (HR-OCT) [22].

In terms of managing OSSN, there are numerous therapeutic options available, based on disease factors, patient factors, and, most crucially, economic considerations [23]. There has been compelling debate on the best therapeutic approach, particularly in the recent decade [23]. Off-label use of topical pharmacotherapeutic agents for ophthalmic administration, including mitomycin-c (MMC) [24,25,26,27,28,29,30,31], 5-fluorouracil (5-FU) [32,33,34,35,36,37], and interferon alpha-2b (IFNa-2b) [38,39,40,41,42,43,44,45], as well as the less commonly used anti-vascular endothelial growth factor (anti-VEGF) agents [8,46,47,48,49,50,51,52], antiviral agents (cidofovir) [8,46,47,48,49,50,51,52], and other modalities, such as photodynamic therapy [8,46,47,48,49,50,51,52], brachytherapy [53], and proton-beam radiotherapy [54], have increased the utilisation of non-surgical OSSN treatment options [55]. Nevertheless, clear margin surgical excision with marginal cryotherapy appears to be the gold standard of treatment [23,24,25]. Conservative and surgical treatments for SCC are believed to be clinically comparable [26], and the treating physicians must assess their patients’ circumstances before deciding on the best course of action [56].

There is an ongoing debate regarding the best treatment approach for ocular surface squamous neoplasia (OSSN), with surgical excision being the established standard of care. However, advancements in medical and conservative therapies have led to their increased use in ophthalmology. Despite these developments, there remains no clear consensus or official guidelines on the optimal treatment regimen for OSSN.

Therefore, the primary purpose of this research is to investigate the existing treatment modalities for OSSN in order to identify the outcomes for optimal disease management in terms of resolution rates, recurrence rates, complications, cost-effectiveness, and patient compliance.

### 1.1. Histopathology

On histological examination, invasive SCC is characterized by malignant squamous cells that transgress through the basement membrane and grow within the stroma [57,58]. The majority of primary malignant tumors of the conjunctiva, specifically SCC, appear in the interpalpebral area near the limbus [57,58]. SCC lesions can start on the conjunctiva and progress to the cornea. These lesions might appear as gelatinous masses, raised leukoplakic masses, nodules, or flat opalescent layers on the cornea [2,9,58].

Diagnosis may be established through clinical examination alone [2,9,58]. Furthermore, slit-lamp examination with special stains, such as Rose Bengal, lissamine green, and methylene blue, can be employed on devitalized squamous cells to aid in OSSN diagnosis [2,9,58].

SCC lesions can spread intraorbitally through surrounding tissues, extraorbitally via regional lymph nodes, and, in rare cases, metastasize to other organs [59]. Based on histopathologic findings, conjunctival squamous neoplasia is classified as carcinoma in situ (CIN grade 1–3), affecting only the epithelium, or as invasive SCC, which invades the basal membrane [2,52]. Advanced SCC lesions rarely penetrate the corneoscleral lamella, access the anterior chamber, or breach the orbital septum to invade orbital tissues [58]. Mucoepidermoid carcinoma, a less common and more aggressive form of conjunctival SCC, refs. [2,9], occurs in elderly patients and has a yellow cystic component due to mucous-secreting cells [10].

The American Joint Committee on Cancer’s (AJCC) eighth edition recently evaluated the classification of conjunctival squamous neoplasia (Table 1) [60]. This classification method is based on two key tumor features: the depth of invasion, which requires histopathologic evaluation, and the size and extent of the neighboring tissues implicated (e.g., cornea, lid, orbit), which can be clinically recognized and studied using imaging techniques. Tumor staging is determined using the TNM (Tumor, Node, Metastasis) definitions outlined in the 8th AJCC recommendations, with T representing the characteristics of the primary tumor, N describing the involvement of regional nodes, and M describing the spread of distant metastasis [61]. T classification is based on tumor size (≤5 mm or >5 mm) and invasion of neighboring structures, including the fornix, plica semilunaris, caruncle, eyelid lamellae, intra-orbital tissues, sinus bones, and brain [33,60,61].

### 1.2. Risk Factors and Pathogenesis

The pathogenesis of SCC through UV radiation is linked to the p53 gene, ref. [62], which codes for a regulatory protein complex involved in carcinogenesis due to mutations triggered by UV radiation [63]. Male gender and older age, with a reported mean presentation age of 56 years, are also implicated in the pathogenesis of the disease [64]. Xeroderma pigmentosum and other genetic causes have been associated with tumor formation and OSSN [65]. Other factors include smoking, immunosuppression, ocular surface injury, exposure to chemicals (petroleum products, beryllium, trifluridine, arsenic) [66], and vitamin A deficiency [8,67]. Several other mutagenic factors at the molecular level have been attributed to the pathogenesis of conjunctiva SCC, such as the telomerase-reverse-transcriptase (TERT) gene promoter [68], A-disintegrin-and-metallopeptidase-domain-3 (ADAM3), especially in high-grade lesions [69], and the overexpression of matrix-metalloproteinase-9 (MMP-9), matrix-metallopeptidase-11 (MMP-11) [70], and clusterin, which is associated with epithelial carcinogenesis in the ocular surface [71]. There have also been reports of association with the human papillomavirus (HPV), serotypes 16 and 18 [7]. However, a study in India indicates that there still lies some controversy in the connection of HPV with OSSN [72]. Higher occurrence is observed in immunosuppressed patients with HIV or AIDS, which accounts for the higher incidence of the disease in afflicted younger women residing in Africa [58,73].

### 1.3. Diagnosis

Anterior segment tumors, including conjunctival OSSN, are commonly diagnosed and monitored using slit lamp examination, serial photography, and ultrasound biomicroscopy (UBM), with the aid of specialized vital dyes, like Rose Bengal, methylene blue, and toluidine blue [22,59,74]. Rose Bengal dye, an iodinated fluorescein derivative, stains apoptotic and metabolically inactive epithelial cells bright pink, aiding in identifying neoplasms [22]. Differential diagnoses for OSSN include pterygium, pinguecula, corneal pannus, vitamin A deficiency, Salzmann’s nodular degeneration, pyogenic granuloma, papilloma, and nevi, especially in patients with complexion-associated melanosis [22,59,74]. Other neoplastic lesions that may masquerade as OSSN include sebaceous cell carcinoma, amelanotic melanoma, conjunctival lymphoma, and keratoacanthoma [50]. For confirmation, histopathological evaluation via biopsy is the standard, ref. [10], though less invasive methods, like impression or exfoliative cytology, in vivo confocal microscopy (IVCM), and high-resolution optical coherence tomography (OCT), are used, each with pros and cons [48].

Cytology is minimally invasive but limited in depth assessments [20,74], while IVCM and OCT provide detailed imaging for initial diagnosis, follow-up, and distinguishing between epithelial and subepithelial lesions [20,75,76]. HR-OCT, or “optical biopsy”, offers high-resolution imaging to predict tumor margins and reduce recurrence after excision, making it valuable in OSSN diagnosis and management [77,78,79].

**Classic Findings on HR-OCT for OSSN**
  1. Thickening of the epithelium with a hyper-reflective character [77,78,80].
  2. Abrupt transition from damaged to normal epithelium [77,78,80].
  3. Demarcation line between the lesion and the normal epithelium. This is commonly more evident in superficial lesions, whereas in more invasive lesions, this may not be distinguished [8].

## 2. Methods of Management

The management of OSSN lesions falls into two main categories: surgical and conservative. Although conservative treatments have advanced, complete surgical removal with adjuvant cryotherapy still remains the preferred method of treatment [81]. Despite the lack of international guidelines, conservative approaches, including topical chemotherapeutic agents like mitomycin-C (MMC), 5-fluorouracil (5-FU), and interferon-a2b (INFa2b), are gaining popularity due to comparable outcomes with surgery [8,46,47,48,49,50,51,82,83,84]. Antiviral drugs (cidofovir), anti-VEGF agents (bevacizumab, ranibizumab), photodynamic therapy, and radiotherapy have also shown success, with combination therapies often used to optimize disease control [8,48,53,84].

### 2.1. Surgical Treatment

The surgical technique used for the treatment of conjunctival SCC lesions differs with the location of the tumor [85]. The majority of primary malignant conjunctival tumors, such as SCC, tend to emerge in the interpalpebral region, specifically near the limbal margin. SCC lesions are preferably managed by excisional biopsy [81,85]. If the lesions occupy the fornix, they can be entirely resected, and the remaining edges of the conjunctiva can be reconstructed and sutured with absorbable sutures as in primary wound closure [13]. In order to prevent adhesions and the creation of symblepharon, fornix deepening sutures or a symblepharon ring can be used. In case of large conjunctival defects, a mucous membrane graft can be imported and is considered a favorable option [86,87]. Limbal neoplasms can reportedly spread through the corneal epithelium and sclera into the anterior chamber or even intraorbitally through adjacent tissue involvement [88]. Therefore, it is strongly advised to remove a thin portion of the underlying sclera to prevent cancerous cell seeding [8]. The technique, which is often used for limbal SCC lesion removal, is partial lamellar sclerokeratoconjunctivectomy with primary wound closure [8]. The operation is performed under a microscope [83]. The surgical field should be left dry by avoiding the use of a balanced salt solution during the procedure in order to ensure cancer cell adherence to the resected specimen and prevent them from spreading throughout the ocular surface [13,89]. During the operation, it is crucial for the surgeon not to touch the tumor with their surgical instruments. This technique is also known as the Shields “no-touch technique” [2,4,13,90].

**Figure 2:**
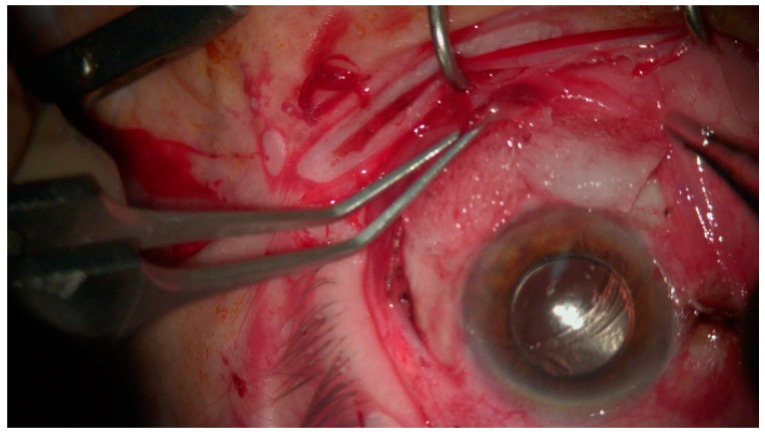
Use of MMC after tumor excision.

Introduced by Shields et al. in 1997, this method applies to tumors with a basal diameter of 15 mm or less or tumors with limbal involvement of four clock hours or less [9,13]. The tumor is excised at 4–5 mm macroscopically clear margins, as recommended by Shields et al. [2], although some authors advocate a narrower margin diameter of 3–4 mm [91]. The main principle of the no-touch technique is that the surgeon should not touch the cancerous lesion at any point during the operation in order to avoid microscopic seeding of the disease [2]. In addition, cancerous cells can spread along the basal layer of the conjunctiva, leading to microscopic positive margins, which fail to be detected intraoperatively [92]. Some authors have subjected the use of preoperative OCT to assist with the delineation of the full tumor perimeter and subsequent complete tumor excision (Figure 2) [80].

**Figure 3:**
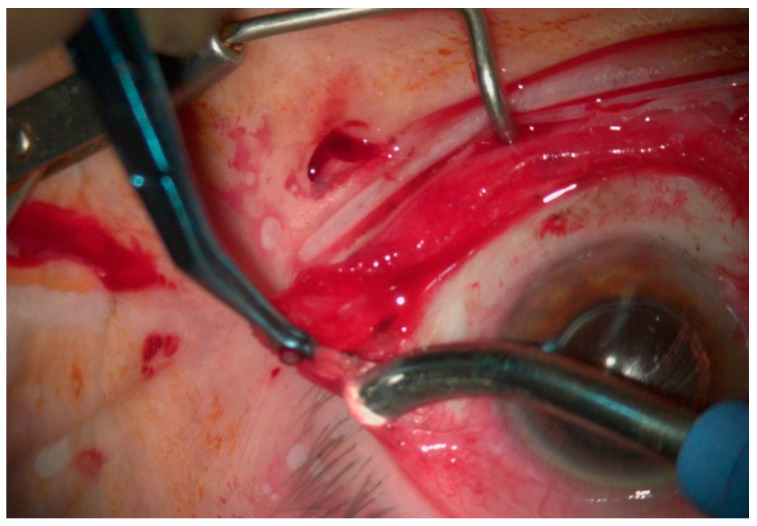
Cryotherapy at the conjunctival edges after excision of OSSN.

Adjuvant treatment with intraoperative MMC or 5-FU alongside marginal cryotherapy has been used to reduce the possibility of disease recurrence [62,93]. The process of post-excisional cryotherapy is achieved in two freeze-thaw cycles at the conjunctival margins and at the base of the excised tissue once the macroscopic tumor-free margins are confirmed [24,89]. Cryotherapy aids in the eradication of residual tumor cells at the excision site through the thermal destruction of cells and blood vessels (Figure 3) [24,89]. The instructed rate of cryoprobe freeze is 2 mm around the probe for the conjunctiva and 1 mm for the limbus in a pulsated rather than a continuous mode [24,89]. It is also advised that the conjunctiva be lifted from the globe during the freeze pulses and cryotherapy be applied to the inner edge of the tissue to protect the underlying sclera [24,89]. The adverse effects of cryotherapy include anterior uveitis, cataract, hypotony, corneal neovascularization, and hemorrhage [67,93].

Under certain circumstances, a modified technique, Mohs micrographic excision, also used to treat skin cancer, may be applied in order to obtain tumor-free margins at the microscopic level [94]. In the case of extensive conjunctival resection, ocular surface repair can be dealt with by the use of a mucous membrane graft or amniotic membrane graft for remodeling the remaining tissue deficit [2,4,90]. Corneal compensation is treated with keratoepitheliectomy using absolute alcohol applied on the involved area for 1 min before the lesion is completely removed at 2 mm clear margins [90]. Primary limbal cell transplantation has been proposed for extensive SCC lesions concerning the limbal area in order to prevent limbal stem cell deficiency [88].

Despite the rapid resolution of the disease, surgical excision alone, even with macroscopically clear margins, is often associated with high recurrence rates due to the presence of microscopic disease beyond the edge of the excised margins [92,95]. The reported recurrence rates range from 0 to 56% in the first two years postoperatively [51,89,96]. Furthermore, the likelihood of recurrence is still present, with a frequency of 33%, despite the presence of clear microscopic margins on pathology specimens. There is evidence that post-excisional adjuvant topical chemotherapy can reduce recurrence rates. MMC is the preferred post-excisional adjuvant agent [85,95,97].

**Figure 4:**
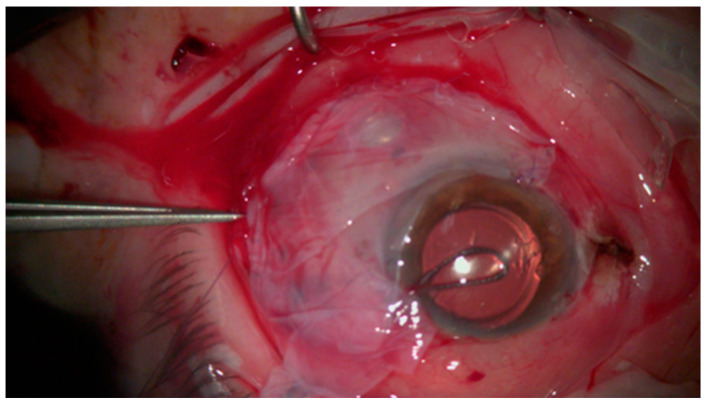
Suturing the AMT onto host conjunctiva.

The most common complications of surgery are limbal stem cell deficiency (LSCD) and symblepharon formation [98]. LSCD can be the result of extensive removal of SCC lesions, which occupy ≥ 6 clock-hours of the limbus, whereas symblepharon can result from the excision of large conjunctival lesions, despite the use of amniotic membrane grafts [13]. However, these complications can be prevented by minimizing scarring and inflammation at the site of resection with the use of amniotic grafts for larger wounds and primary wound closure for smaller wounds (Figure 4) [86,87,99,100]. Concomitant limbal epithelial transplantation has also been used for the prevention of LSCD [88]. Additional uncommon complications include postoperative infections, pyogenic granulomas, and injury to the retina and sclera as a result of excessive cryotherapy [101]. Surgically induced scleral necrosis (SINS) is a severe condition that may manifest after surgery, resulting in scleral dissolving and perforation [56].

Surgical excision of primary OSSN lesions with marginal intraoperative cryotherapy has been the commonest treatment mode, with cryotherapy playing an essential role in reducing the likelihood of disease return [2,22,25,63], However, a “standard of care” survey conducted in 2013 proved that topical medications are gradually becoming common practice with equivalent therapeutic results to surgery [55].

The use of topical chemotherapy can be administered in the form of monotherapy or in combination with surgical removal of the lesion, either preoperatively or postoperatively [31,44,101]. Evidently, intraoperative marginal cryotherapy can reduce the risk of recurrence [24,89]. Other means used to decrease recurrence rates at 0–21% are postoperative topical MMC and postoperative topical IFNa-2b in patients with positive margins [85,97,102,103]. In fact, adjuvant IFNa-2b has been proven to decrease recurrence rates in patients with positive surgical margins to the level of patients with tumor-free margins [85]. The application of adjuvant post-excisional proton radiotherapy has also been successful in the reduction in recurrence rates in patients with conjunctival SCC [51].

### 2.2. Medical Treatment

Medical treatments for OSSN have gained popularity, with topical medications serving as primary therapy [30,37,104] or auxiliary to surgery [55]. Adjunctive chemotherapy is administered preoperatively for chemoreduction, ref. [105], intraoperatively at the excised margins, or postoperatively to reduce the risk of recurrence [104]. The growing popularity of medical treatments in OSSN can be attributed to the necessity for more convenient in-office therapeutic options, the prevention of serious complications associated with surgery, and the development of less invasive diagnostic modalities, such as in vivo confocal microscopy, HR-OCT, and impression or exfoliative microscopy. The most commonly used chemotherapeutic agents are MMC [24,25,26,28,29,30,31,93,97,106,107,108], 5-FU [33,34,35,36,37,109,110], IFNa-2b [13,40,41,42,43,44,91,95,106,110,111,112,113,114,115,116,117,118,119], and, to a lesser extent, anti-VEGF agents, such as bevacizumab and ranibizumab for conjunctival SCC [120,121,122,123] and cidofovir in HPV-affected patients with OSSN as the most frequently used chemotherapeutic agents [23,56,124].

The efficacy of chemotherapeutic medications as monotherapy in the treatment of primary OSSN lesions has been demonstrated to be comparable to that of surgical treatments in terms of disease resolution and recurrence rates [24,26,30,101,102].

Despite the lack of a universal consensus in the management of OSSN lesions, Karp et al. suggested that larger lesions occupying >4 clock hours of the ocular surface or ≥5 mm (AJCC), as well as multifocal and recurrent lesions, can be effectively treated with topical chemotherapeutic medications, such as MMC, 5-FU, and IFN [8,115]. The main disadvantages of the medical care of OSSN are the prolonged periods required until disease remission, leading to difficulties related to the patient’s compliance with medication [104].

#### 2.2.1. 5-Fluorouracil

5-Fluorouracil (5-FU) is a fluoropyrimidine antimetabolite structurally similar to thymine and uracil, which inhibits RNA and DNA functions during the S-phase of mitosis. It is widely used for cancers, such as colorectal and breast cancer, ref. [125], and also effectively treats ocular surface squamous neoplasia (OSSN) through topical chemotherapy—either as monotherapy, neoadjuvant, ref. [32], or adjuvant therapy post-surgery [126,127,128].

5-FU enters cells like uracil and is converted into active metabolites that impair RNA synthesis and thymidine synthase, leading to DNA damage and cell death. Its minimum inhibitory concentration for fibroblasts is 0.35 mg/mL, and studies in rabbits show intraocular penetration without retinal toxicity. It is generally well tolerated compared to other agents, like mitomycin C (MMC), with fewer side effects, such as ocular pain, conjunctival hyperemia, and occasional corneal complications. Regular artificial tears and corticosteroids are recommended to manage side effects, with cyclic administration (3–4 days on, one month off) for disease resolution [129].

5-FU has fewer adverse effects than MMC but more than interferon alpha-2b (IFNa-2b), as it affects normal and cancerous cells alike. It has high efficacy (85–100%), ref. [32], with low recurrence rates (6–14% as monotherapy, refs. [32,37], 1.1–4.3% as adjunctive) [127]. It has also shown success in refractory cases after MMC treatment [130] (Table 2).

5-FU is cost-effective, priced at approximately $75 per bottle in the US, making it cheaper than MMC and IFNa-2b. Despite needing compounding, it remains stable for 3 weeks, can be stored at room temperature, and is user-friendly [104].

Studies report high efficacy: 82% complete tumor resolution in a 44-eye study, with a 9% recurrence at two years. Other studies show similar results, with 96% tumor resolution compared to 81% for IFNa-2b. However, 5-FU treatment has a longer duration and more surface toxicity compared to surgery or MMC, which may affect patient compliance [101,128] (Table 2).

#### 2.2.2. Mitomycin-C

Mitomycin-C (MMC) is an alkylating agent with antitumor, antibiotic, and antimetabolite properties. It originates from the broth of *Streptomyces caespitosus* cultures and belongs to the family of antitumor quinolones [131]. MMC induces DNA damage through two primary pathways: by generating free radicals, which damage DNA and proteins in aerobic conditions, or through DNA alkylation in anaerobic conditions [131]. MMC’s electrophilic nature facilitates the formation of carbonium ion intermediates, which bind covalently to DNA and other nucleophiles [131]. This activation occurs via cytochrome p450 reductase, leading to DNA cross-linking, primarily between adenine and guanine residues, which inhibits DNA synthesis and disrupts cell mitosis, causing cell death [131]. Notably, a single DNA cross-link can be lethal to bacterial cells. MMC primarily acts during the late G1 and S phases of the cell cycle, though alkylation can occur at any stage [131].

Due to its non-cell-cycle specificity, MMC affects both cancerous and rapidly dividing normal cells. Its cytotoxicity is partially attributed to glutathione depletion within cells. Despite these challenges, MMC has shown effectiveness in treating a range of anterior segment disorders, including glaucoma, pterygium, conjunctival intraepithelial neoplasia, and corneal neoplasia, with favorable outcomes [131].

In the context of conjunctival and corneal SCC, MMC has been explored as a topical treatment. Studies report its use as a preoperative chemo-reductive agent [105,108,132], intraoperatively, and postoperatively as an adjunct to surgery [25,29,93,97,127,133]. Preoperative MMC has been used to reduce tumor size in minimally elevated SCC lesions, with recurrence rates as low as zero [105]. Intraoperatively, MMC has been applied to the subconjunctival surface using sponges soaked in 0.02% to 0.04% solutions [29]. Postoperative use typically involves MMC eye drops (0.02%), administered three times daily for two-week cycles, often with punctal occlusion to prevent systemic absorption [108].

Numerous studies suggest MMC reduces recurrence rates when used postoperatively, even in cases with positive surgical margins [24,26,28,29,30,31,106,107,108,113]. For example, a retrospective review showed no recurrences over a mean follow-up period of 49 months when MMC was applied following surgical excision and cryotherapy [93]. These findings highlight MMC’s potential in lowering recurrence rates when used as an adjunct to surgery, compared to surgery alone, where recurrence rates can be as high as 5–33% over 15 years of follow-ups [92,96].

Several studies emphasize the benefits of MMC for ocular surface squamous neoplasia (OSSN) lesions, showing high resolution rates (92–100%) and low recurrence rates [106]. For instance, a retrospective study by Kusumesh et al. reported a 92% resolution rate in OSSN patients treated with MMC (0.04%) drops four times daily, with a median resolution time of 1.5 months and no recurrences at 18 months [106]. Other studies confirmed recurrence rates between 0% and 22%, with recurrences typically observed after 24 months [30,107,108]. The standard therapeutic regimen for MMC involves cycles of one week of application followed by 1–2 weeks of cessation, with a maximum of three cycles [26,30,48,95,97,105]. When used as monotherapy, MMC shows success primarily with intraepithelial lesions and is less effective for more invasive conjunctival SCC [30].

MMC, while generally well tolerated, presents some risks. Complications range from mild side effects, like dry eye, punctate keratitis, and chemosis, to more serious issues, such as scleral melting, corneal melting, iritis, and cataract formation [30,106,107,108,134,135]. These adverse effects are more pronounced when MMC is applied for extended periods or at higher concentrations (e.g., 0.1%) [136]. Additionally, topical MMC may cause allergic reactions, conjunctival hyperemia, punctate corneal epitheliopathy, and punctal stenosis [27]. Punctal stenosis, which can lead to chronic tearing (epiphora), occurred in 14% of patients in a study involving OSSN treatment with MMC [27]. To mitigate the adverse effects, physicians may prescribe preservative-free artificial tears or corticosteroids [137].

Despite its potential side effects, MMC remains a valuable option, especially as a preoperative chemo-reductive agent or postoperative adjunct therapy [136]. Studies show that neoadjuvant MMC reduces tumor size, facilitating smaller excisions and yielding better surgical outcomes [105]. Moreover, its affordability compared to other therapies (e.g., interferon-alpha-2b) [106] makes it a viable option for many patients. In the U.S., for instance, MMC costs between $100 and $200 per bottle, with out-of-pocket costs being significantly lower than alternatives, such as interferon-alpha-2b, which can range from $240 to $600 per month [104].

However, MMC should be used cautiously, especially when dealing with thicker or more invasive SCC lesions, for which surgical excision remains the preferred treatment. While MMC’s toxic effects on the ocular surface render it less preferable as a first-line treatment for some clinicians, it has been proven to significantly reduce recurrence rates when applied postoperatively, even in cases with positive margins [105,132].

Comparing MMC with other topical chemotherapy agents, like 5-fluorouracil (5-FU) and interferon-alpha-2b (IFNa-2b), reveals that MMC has a shorter treatment period (1.5 months vs. 3.5 months for IFNa-2b) but a higher toxicity profile. Both 5-FU and IFNa-2b display similar efficacy in treating OSSN lesions, with resolution rates of 92% for MMC and 89% for IFNa-2b [106]. Recurrence rates are comparable across agents, though IFNa-2b is associated with fewer side effects [106] (Table 3).

In conclusion, MMC is a well-established treatment for various ocular surface disorders, including conjunctival and corneal SCC [107,108,138,139]. While it is not recommended as monotherapy for SCC, MMC serves as a valuable preoperative and postoperative therapy [97,133]. Its relatively low cost, ease of preservation, and manageable side effects make it a practical option in clinical practice [55]. However, clinicians must balance its benefits against the risk of adverse effects and consider alternative therapies like IFNa-2b or 5-FU, particularly in patients with less invasive OSSN lesions or those who are sensitive to MMC’s toxic profile [113] (Table 3).

#### 2.2.3. Interferon-Alpha

Interferons (IFNs) are a class of cytokines, specifically glycoproteins, that play a significant role in immunomodulation, exhibiting anti-proliferative, anti-angiogenic, cytotoxic, and immunostimulatory effects [140,141,142]. Their antitumor action is linked to their ability to bind to immune cell surface receptors, inhibit protein synthesis, and activate pathways that promote antiviral and antitumor responses [140,141,142]. IFNs have an impact on tumor cells through mechanisms like cell cycle inhibition (by halting cyclin-dependent kinases), promoting apoptosis, and inhibiting angiogenesis, which is crucial for tumor growth [140,141,142]. They also aid in the regulation of immune surveillance, particularly influencing CD8+ cytotoxic T cells, which contribute to the targeting of tumor cells [140,141,142].

IFNs are classified into two main types: Type I (which includes IFN-alpha) and Type II [38]. IFN-alpha (IFNa) is particularly noteworthy for its use in treating malignancies such as ocular surface squamous neoplasia (OSSN) [38]. There are two main forms of IFN-alpha in clinical use: interferon alpha-2a (IFNa-2a) and interferon alpha-2b (IFNa-2b) [106]. IFNa-2b, a recombinant form, is commonly used in OSSN treatment either as ophthalmic drops or through subconjunctival or perilesional injections [106]. The two IFNa variants differ by an amino acid at position 23, where IFNa-2a has lysine, and IFNa-2b has arginine [143].

In OSSN treatment, IFNa-2b has shown efficacy, especially when administered topically or by injections, with the latter offering faster tumor resolution [42,43,114]. IFNa-2b treatment also enhances immune responses, making it suitable for immunocompetent patients, while alternative agents like 5-FU or MMC may be preferable in immunosuppressed patients [144]. IFNa is employed as monotherapy, neoadjuvant chemotherapy for tumor shrinkage, or adjunctively after surgical excision to reduce recurrence [40,43,104,114,145].

Topical IFNa-2b has gained popularity for its minimal toxicity and safety profile in treating OSSN, often achieving high success rates (81–100% resolution) with low recurrence rates (0–5%) [44,106,110,111,112,146]. The standard topical dose is 1 million international units (MIUs) per milliliter, administered four times daily (QID), with a mean time to resolution of approximately four months [23,56,104,114,147]. Injections of IFNa-2b (typically 3 million IU per mL) are often given weekly until tumor resolution, and higher doses, like 10 million IU, have been used with success in some cases [43,44] (Table 4).

Pegylated IFNa (PEG-IFNa), a modified form with a longer half-life, offers another treatment option with less frequent administration [148]. PEG-IFNa-2a and PEG-IFNa-2b have been used in OSSN treatments, particularly in patients requiring prolonged therapeutic activity [82,148,149,150,151,152]. Both topical and injected forms have shown efficacy, with reported success in achieving tumor resolution [145,149] (Table 4).

In comparing topical drops to injections, injections offer a faster resolution, more direct delivery to the tumor site, and greater assurance of compliance, as they bypass the need for patient-applied topical drops [43]. Additionally, injections may be more readily available and covered by insurance in some regions [8,42,43,82,114].

In summary, IFNa, especially IFNa-2b, is a valuable treatment option for OSSN, offering multiple administration routes, high efficacy, and a favorable safety profile, particularly in immunocompetent patients [150] (Table 4).

## 3. Other Methods of Management

### 3.1. Cidofovir

Cidofovir is a monophosphate nucleotide analogue that functions as an antiviral agent primarily used to treat cytomegalovirus (CMV) [151,152]. Its mechanism of action targets double-stranded DNA viruses, such as CMV and human papillomavirus (HPV) [151,152]. Given HPV’s role in oncogenesis, particularly subtypes 16 and 18, cidofovir has been investigated for its antitumor properties in treating ocular surface squamous neoplasia (OSSN), especially in cases refractory to other treatments, like interferon alpha-2b (IFNa-2b) [151,152].

Topical cidofovir has shown efficacy in the short- and medium-term treatment of OSSN, particularly in HPV-related lesions, with case reports highlighting its success in high-grade intraepithelial neoplasia and conjunctival squamous cell carcinoma in situ [151,152]. In one case, topical cidofovir (2.5 mg/mL, three times daily) resulted in complete tumor clearance after four weeks, with no recurrence after 18 months [151,152]. Another case saw successful treatment of diffuse conjunctival squamous cell carcinoma in situ with a more intensive regimen (one drop every two hours, gradually reducing to three times daily over six weeks), followed by surgical excision and cryotherapy. No recurrence was observed after two years of follow-up [151,152].

However, there are some side effects associated with cidofovir use, including cicatrization of the punctum, ocular irritation, conjunctival hyperemia, and pain, which typically subside after discontinuation of therapy [151,152]. Despite these promising results, the cost of treatment, approximately $300–400 for a 5 mL vial in the U.S., may be a limiting factor for some patients [151,152].

The antitumor potential of cidofovir, particularly in HPV-related OSSN, warrants further investigation, as there is a lack of large-scale, comparative clinical trials exploring the drug’s effectiveness and its correlation with HPV infections [151,152]. Further research is necessary to better understand cidofovir’s role in the treatment of OSSN and its potential as a therapeutic alternative for patients resistant to traditional therapies, like IFNa-2b [151,152].

### 3.2. Anti-Vascular Endothelial Growth Factor

Anti-vascular endothelial growth factor (anti-VEGF) agents, such as bevacizumab and ranibizumab, are monoclonal antibodies that inhibit blood vessel formation (angiogenesis) and have been explored as treatments for conjunctival squamous cell carcinoma (SCC), particularly in ocular surface squamous neoplasia (OSSN) [153,154]. Bevacizumab is a full anti-VEGF antibody, while ranibizumab is an antibody fragment, and both have been used in the form of topical drops and intralesional injections in treating these lesions [121,123,153,155].

#### 3.2.1. Bevacizumab in OSSN

Several studies have examined the use of bevacizumab in OSSN:

- In a small study, topical bevacizumab (5 mg/mL, four times daily) was used for eight weeks in six patients. Two patients achieved complete resolution, while the others showed significant tumor size reductions (42–100%) [155].
- In terms of dosage, a subconjunctival/perilesional injection of 2.5 mg/0.1 mL weekly for two weeks has been shown to produce some response [123]. However, a lower dose of 1.25 mg/0.05 mL in cases refractory to topical chemotherapy was insufficient to treat the disease [122].
- Despite these promising outcomes, bevacizumab appears to impede the healing of corneal epithelial defects, which could be a concern for patients [122,123,155].

#### 3.2.2. Ranibizumab in OSSN

Ranibizumab, a smaller antibody fragment compared to bevacizumab, has also been investigated as follows:

- A study on subconjunctival ranibizumab (0.5 mg) administered monthly or twice monthly demonstrated complete resolution in 34% of patients and partial resolution in 66%, with no recurrences after six months of follow-ups [121].
- Though these results are promising, larger studies are required to validate the effectiveness of ranibizumab in OSSN [122,123,155].

#### 3.2.3. Limitations and Future Research

The anti-VEGF agents show promise, particularly for conjunctival lesions, but their role in corneal lesions is less clear [121]. Although initial findings suggest that both bevacizumab and ranibizumab may have utility in treating OSSN, especially recurrent or refractory cases, the literature remains limited [122,123,155]. Further research and larger-scale clinical trials are necessary to establish these agents as standard treatment modalities in OSSN management [122,123,155]. Additionally, the potential for delayed corneal epithelial healing with bevacizumab should be carefully considered in clinical practice [122,123,155].

### 3.3. Radiotherapy

Radiotherapy is a key treatment for OSSN and conjunctival SCC. The two main types are external beam radiotherapy (EBRT) and brachytherapy [54]. Proton and electron EBRT are preferred for sparing nearby healthy tissues [54]. EBRT has been used for large tumors, including those with intraocular invasion, often preventing enucleation, with good outcomes and minimal side effects [54,156,157]. Postoperative proton beam therapy has been shown to reduce disease relapse in conjunctival SCC [51], and it has also been used palliatively in HIV-positive patients with advanced intraocular invasion [158].

Brachytherapy is typically an adjunct to surgery, using isotopes like strontium-90 (Sr-90), iodine-125 (I-125), and ruthenium-106 (Ru-106) [159]. Radioactive plaques target the tumor while protecting the surrounding tissues [159]. Studies have shown promising results, including tumor control even with positive margins and minimal side effects, like dry eye or astigmatism [160].

## 4. Photodynamic Therapy (PDT)

PDT combines the photosensitizer verteporfin with a laser to induce tumor necrosis [50]. A pilot study showed 100% tumor resolution in conjunctival SCC cases, with no recurrences in follow-ups [50]. However, the high cost, lack of availability, and need for specialized training limit its use [161]. PDT has also successfully treated large OSSN lesions with no recurrence after 13 months [162].

Both radiotherapy and PDT show promising results for OSSN and conjunctival SCC, though further studies are needed to solidify their roles.

## 5. Conclusions

Conjunctival squamous cell carcinoma (SCC), a subset of ocular surface squamous neoplasia (OSSN), includes various stages of disease, from corneal intraepithelial neoplasia (CIN) to invasive SCC [2]. Although treatment options lead to favorable outcomes, untreated conjunctival SCC can progress to more severe, potentially sight-threatening conditions, such as limbal stem cell deficiency, orbital invasion, and distant metastasis [9]. It has a mortality rate of 8–24%, and orbital invasion occurs in about 10% of cases [163,164].

### 5.1. Risk of Recurrence and Progression

Recurrence of conjunctival SCC is more likely in patients with larger tumors, older age, positive surgical margins, HIV infections, a higher tumor grade, the presence of feeder vessels, and a higher proliferation index [2,9,13]. Additionally, HIV-positive patients often present with more aggressive disease, which may increase the likelihood of recurrence and metastasis [5,9,10,165,166].

### 5.2. Diagnosis

While non-invasive diagnostic methods, like impression cytology, in vivo confocal microscopy (IVCM), and high-resolution optical coherence tomography (HR-OCT) can help detect lesions, excisional biopsy remains the gold standard for diagnosing SCC [8,16,46,47,48,49,50,51,52,81,82,83,84,101]. HR-OCT is particularly useful in differentiating invasive from in situ disease by identifying thickened, hyperreflective epithelium (>120 microns) [77,78,80]. Exfoliative and impression cytology are less reliable, as they are often unable to distinguish between superficial and invasive lesions [19,20,76,167,168].

### 5.3. Surgical Treatment

Surgical excision is the mainstay of treatment for small lesions (less than 4 clock hours or 5 mm) and is performed using the “no-touch” technique with cryotherapy to the margins to reduce the recurrence risk [23,81]. Surgery offers quick resolution and is recommended in patients with systemic contraindications to topical therapy [8,16,46,47,48,49,50,51,52,81,82,83,84,101]. However, surgery may have drawbacks, such as cost, risk of complications, and recurrence, particularly in larger or recurrent tumors [23,49,81,127,169].

### 5.4. Topical Chemotherapy

Topical chemotherapeutic agents, like mitomycin-C (MMC) [24,25,26,28,29,30,31,95,107,108,127], 5-fluorouracil (5-FU) [32,34,35,36,37], and interferon alpha-2b (IFNa) [13,38,39,40,42,43,44,45,106,112,114,115,116], are often used to complement surgery. These medications can be administered before surgery (neoadjuvant) to shrink the tumor [31,83,84], intraoperatively [25,31], or postoperatively [108,124,129,170] to reduce recurrence. Chemotherapy is also useful for larger tumors (>4 clock hours or 5 mm), multifocal, or nasally located lesions where complete excision may be challenging [13,28,45,49,101,119,169,171].

### 5.5. Radiotherapy

For more advanced or invasive conjunctival SCC, particularly those with corneo-scleral involvement, radiotherapy is an effective alternative [159]. Proton and electron beam radiotherapy can target the tumor while sparing healthy tissues, like the cornea, retina, and lens [54]. Brachytherapy using radioactive isotopes (e.g., strontium-90, iodine-125, and ruthenium-106) is often employed as an adjunct to surgery for localized lesions [159]. Both techniques have been shown to achieve good tumor control with minimal side effects, making them viable options in cases where surgery is not feasible or as palliative therapy [160,162].

### 5.6. Photodynamic Therapy (PDT)

PDT involves the use of verteporfin, a photosensitizing agent, combined with a diode laser to generate oxygen radicals that destroy the tumor [162]. Although effective in small studies, PDT is limited by its high cost and availability, as well as the need for specialized training [50,161,162].

### 5.7. Patient Considerations

Patient factors play a significant role in choosing between surgical and medical treatments [47,48]. These include the costs of therapy, access to healthcare, compliance with the longer duration of topical chemotherapy, and socioeconomic factors, such as transportation or time off work. In the U.S., surgical excision can be costly, while topical chemotherapies, particularly IFNa-2b, also represent significant expenses [8,82]. Subconjunctival injections may be more cost-effective and require fewer visits, making them an attractive option for patients struggling with compliance or financial barriers [8].

In summary, conjunctival SCC management requires a tailored approach based on the size, location, and invasiveness of the tumor, as well as patient-specific factors (Table 5). Surgical excision is preferred for smaller lesions, while topical chemotherapy and radiotherapy are reserved for larger, recurrent, or more invasive tumors. Treatment decisions should balance efficacy, patient compliance, and cost.

Combining TNM staging-based OSSN treatment with considerations for special populations—HIV patients, pregnant women, and infants—requires tailored approaches to address their unique needs while maintaining effectiveness and safety.

HIV Patients
  1. Impact of Immunosuppression:
    ○ HIV-positive patients are at a higher risk for OSSN recurrence due to immune compromise, particularly when CD4 counts are low.
    ○ Tumors in HIV patients may present more aggressively, necessitating more vigilant staging and follow-ups [84].
  2. Preferred Treatments:
    ○ Topical chemotherapy: interferon alpha-2b or 5-fluorouracil is often favored over surgery to reduce complications.
    ○ Highly active antiretroviral therapy (HAART): active antiretroviral therapy has been shown to improve OSSN outcomes by bolstering immune response [119].
  3. Follow-Up:
    ○ Frequent monitoring post-treatment is critical to detect early recurrence.
Pregnant Women
  1. Treatment Modifications:
    ○ Avoidance of teratogenic agents: chemotherapy agents like mitomycin-C and 5-fluorouracil are contraindicated due to potential teratogenic effects [172,173].
    ○ Surgical approach: localized surgical excision with cryotherapy is typically the preferred method for managing OSSN in pregnant women [90].
  2. Monitoring and Timing:
    ○ Treatment is ideally postponed until after delivery if the disease is non-aggressive.
    ○ Multidisciplinary coordination with obstetricians is crucial.
Infants and Young Children
  1. Unique Considerations:
    ○ Rare occurrence in this age group, but tumors may have an aggressive course if present.
    ○ Emphasis on conservative, less invasive treatment options to preserve ocular function.
  2. Treatment Options:
    ○ Surgery is often the first-line treatment, minimizing extensive tissue removal to preserve ocular anatomy.
    ○ Use of topical treatments like interferon alpha-2b has been reported with good tolerance in pediatric cases [115].
  3. Monitoring:
    ○ Regular follow-ups to monitor for recurrence and ensure normal development of ocular structures.
Synthesis of Approaches

In all these populations, the TNM staging system remains central to assessing the extent of disease and guiding treatment. However, the unique physiological and immunological status of HIV patients, pregnant women, and young children requires modifying standard treatments to optimize safety and outcomes. A multidisciplinary team approach—incorporating oncologists, ophthalmologists, obstetricians, and pediatricians—is essential for effective management.

## Figures and Tables

**Table 1:** AJCC 8th edition TNM classification for OSSN [60].

Category	Description
Primary Tumor (T)	
Tis	Carcinoma in situ (confined to the epithelium, no invasion into the substantia propria).
T1	Tumor limited to the conjunctiva without invasion into adjacent structures.
T2	Tumor invades adjacent ocular structures (e.g., cornea, fornix, caruncle, sclera, or globe).
T3	Tumor invades adjacent structures, such as the orbit, sinus, or eyelid.
T4	Tumor demonstrates further invasion into the central nervous system or other regional or distant sites.
Regional Lymph Nodes (N)	
N0	No regional lymph node metastasis.
N1	Regional lymph node metastasis present.
Distant Metastasis (M)	
M0	No distant metastasis.
M1	Distant metastasis present.

Notes: Anatomical divisions: the conjunctiva is divided into bulbar, fornix, tarsal, and caruncle regions for localization. Staging summary: Stage 0: Tis, N0, M0; Stage I: T1, N0, M0; Stage II: T2, N0, M0; Stage III: T3, N0, M0, or any T, N1, M0; Stage IV: any T, any N, M1.

**Table 2:** Dosage regimens of 5-fluorouracil for the treatment of OSSN.

Dose: 1% topical 5-FU QID for 1 week, followed by a drug holiday of 3 weeks	Response rate: 52/54 (96%) Time to response: 6.6 ± 4.5 weeks	Recurrence rate: 6/54(12%), mean of 7.7 ± 9.1 months	Side effects: pain (12, 22%), tearing (12, 22%), redness (11, 20%), eyelid edema (5, 93%), keratopathy (4, 7%), no long-term complication
Dose: 1% 5-FU topical QID for 4 weeks, adjunctive courses administered after 1 month of chemotherapy-free interval	Response rate: 34/41 (83%) Time to response: 11 ± 9 weeks (range: 3–22 weeks)	Recurrence rate: 4/41 (9%)	Side effects: photophobia (20, 51%), conjunctival hyperemia (19, 48%), irritation (17, 43%), pain (14, 36%), superficial punctate keratitis (11, 28%), lid erythema (4, 8%)
Dose: 1% topical 5-FU QID for 1 week, followed by a drug holiday of 3 weeks No punctal plugs	Response rate: 82% (36/44) Time to response: 3.8 cycles	Recurrence rate: 11.1% (4/36)	Side effects: Pain (39%) Tearing (23%) Photophobia (14%) Itching (9%) Swelling (5%) Infection (2%)
Dose: 1% 5-FU topical QID for 4 weeks, adjunctive courses administered after 3 months of chemotherapy-free interval	Response: 22 (100%) Time to response: Not specified Endpoint: Until resolution	Recurrence: 3 (14%)	Side effects: none reported (eye ointment applied to the inferior eyelid to minimize skin contact, and inferior lacrimal punctum occluded)
Dose: 1% 5-FU QID for four days, followed by a 30-day break for 6 cycles.	Response rate: 100% (15/15)	Recurrence rate: 6.7% (1/15)	Side effects: Eyelid skin irritation Conjunctival redness

**Table 3:** Dosage regimens of mitomycin-C for the treatment of OSSN.

Dose: 0.04% drops QID for a week, then 2–3 weeks cessation of therapy for a maximum of 3 cycles.	Endpoint: Until resolution Response: 102 (79.1%)	Recurrence: 15.1%	Side effects: redness, irritation, papillary conjunctivitis, lid swelling, corneal erosion, and punctal stenosis
Dose: 0.4 mg/mL or 0.04% QID for 3 weeks Advised punctal plugs or manual occlusion	Endpoint: Until resolution by slit lamp examination or failure to regress by 6 weeks Response: 24 (92%) Time to response: 121 days (range: 73–169)	Recurrence: Not specified	Side effects: redness, irritation
Dose: 0.02% QID for 2 weeks, followed by 2 more weeks if not resolved. Manual punctal occlusion for 3 min	Endpoint: For 28 consecutive days Response: 18 (100%) Time to response: 28 days	Recurrence: 0 (0%)	Side effects: conjunctival hyperemia, tearing, corneal epithelial erosions (2, 11%)
Dose: 0.04% QID for 1 week and then 1-week pause for a mean of 3 cycles (range 1–4)	Endpoint: Until clinical resolution Response: 10/10 (100%)	Recurrence: 0 (0%) At a mean follow-up time of 22 months	Side effects: redness, irritation, erythema, punctate keratopathy, chemosis.
Dose: MMC 0.4 mg/mL (0.04%) QID in 1-week on and 1-week off cycles Advised punctal occlusion for 5 min	Endpoint: Until clinical resolution or failure to regress after 2 cycles Response: 23 (92%) Time to response: 1.5 ± 0.54 (median 1.5)	Recurrence: 0 (0%)	Side effects: Conjunctival hyperemia (11, 44%), hyperemia with burning sensation (9, 36%), corneal epitheliopathy (3, 12%), photophobia with blepharospasm (2, 8%), punctal stenosis (1, 4%).

**Table 4:** Dosage regimens of interferon alpha-2b for the treatment of OSSN.

Dose: 1 MIU/mL topical IFNα−2b QID	Endpoint: Until clinical resolution or failure to regress within 3 months Response: 22 (92%) Time to response: median: 3.25 m, range: 2–4 m	Recurrence: 0 (0%)	Side effects: intratumoral bleeding (2, 8%), conjunctival congestion (1, 4%), foreign body sensation (1, 4%)
Dose: 1 MIU/mL topical IFN IFNα−2b QID	Endpoint: Until clinical resolution or failure to regress after 2 months Response: 22 (92%) Time to response: median: 3.25 m, range: 2–4 m	Recurrence: 1 (4%) at 18 m	Side effects: conjunctival hyperemia (2, 8%), hyperemia with burning sensation (1, 4%)
Dose: 1 MIU/mL topical IFNα−2b QID	Endpoint: Until biomicroscopic evidence of tumor resolution or until the time a secondary treatment was deemed necessary due to poor response Response: 59 (95%) complete response; 2 (3%) had partial response; additional treatment required for complete response in 7 (11%) Time to response: 5.8 m (median: 5, range: 1–17.8)	Recurrence: 2 (3%)	Side effects: follicular reaction (4, 6%), corneal epithelial defect (2, 3%), irritation (1, 2%)
Dose: 1 MIU/mL topical IFNα−2b QID	Endpoint: Until biomicroscopic evidence of tumor resolution or until the time a secondary treatment was deemed necessary due to poor response Response: 59 (95%) complete response; 2 (3%) had partial response; additional treatment required for complete response in 7 (11%) Time to response: 5.8 (median: 5, range: 1–17.8)	Recurrence: 2 (3%)	Side effects: follicular reaction (4, 6%), corneal epithelial defect (2, 3%), irritation (1, 2%)
Dose: 3 MIU/mL topical IFNα−2b QID	Endpoint: Until 1 month beyond complete tumor resolution then tapered to BD for 2 months, or until failure to regress tumor in 2 subsequent monthly visits Response: 89 (97%); 8 required perilesional IFNα−2b Time to response: 4.64 ± 1.92 months (median: 5, range: 1–10)	Recurrence: Not specified	Side effects: conjunctival hyperemia (4, 4%), follicular reaction (2, 2%), punctate epithelial erosions (1, 1%), chemosis (1, 1%)
Dose: 3 MIU/0.5 mL perilesional subconjunctival IFNα−2b weekly	Endpoint: Until resolution Response: 13 (87%) Time to response: median: 1.4 months, range 0.6–5.7	Recurrence: 1 (7%)	Side effects: stinging and irritation (4, 27%), fever and malaise (5, 33%)
Dose: 3 MIU/0.5 mL intralesional IFNα−2b once weekly	Endpoint: Until resolution Time to response: 6.5 months, range: 4–11 months	Recurrence: 0 (0%)	Side effects: None reported
Dose: 3 MIU/0.5 mL perilesional subconjunctival IFNα−2b once followed by 1MIU/mL topical IFNα−2b QID	Endpoint: Until 1 month after clinical resolution Response: 6 (100%) Time to response: within 6 weeks	Recurrence: 0 (0%)	Side effects: fever and myalgia (2, 33%)

**Table 5:** Treatment for conjunctival OSSN based on the AJCC 8th edition TNM classification.

Primary Tumor (T) Staging: T1: Tumor ≤5 mm, confined to the conjunctiva Surgical: ○ Excisional biopsy with wide margins. ○ Consider cryotherapy to surgical margins to minimize recurrence. Medical: ○ Topical chemotherapy (mitomycin-C, 5-fluorouracil, or interferon-α2b). Adjuvant: ○ Frequent monitoring to detect recurrences early. T2: Tumor >5 mm or involves adjacent structures Surgical: ○ Wide local excision with cryotherapy. ○ Reconstruction, if necessary (e.g., amniotic membrane grafts). Medical: ○ Topical chemotherapy post-surgery to address residual disease. Adjuvant: ○ Imaging to evaluate deeper invasion and monitor high-risk margins. T3: Tumor invades deeper tissues (e.g., eyelid lamellae, intra-orbital tissues) Surgical: ○ Exenteration (orbital contents removal) in extensive cases. Medical: ○ Systemic chemotherapy to control disease progression. Adjuvant: ○ Radiation therapy for non-resectable residual disease or palliation. T4: Tumor invades sinuses, bone, or brain Surgical: ○ Radical surgery (often limited by feasibility or patient status). Medical: ○ Systemic chemotherapy as primary treatment or palliative option. Adjuvant: ○ Radiation for symptom management. ○ Multimodal imaging to guide treatment strategies.
Regional Nodes (N) and Metastasis (M) Staging: N1: Regional lymph node involvement Surgical: ○ Lymph node dissection or biopsy. Medical: ○ Systemic chemotherapy targeting nodal disease. Adjuvant: ○ Radiation to involved nodal basins. M1: Distant metastasis Systemic: ○ Chemotherapy (often platinum-based or immunotherapy) tailored to metastatic disease. ○ Palliative care to address symptoms and improve quality of life.
General Considerations: Imaging: ○ Employ MRI/CT for deeper tissue involvement (T3–T4). ○ Ultrasound for surface tumors (T1–T2). Histopathology: ○ Evaluate invasion depth and margin involvement post-surgery. Interdisciplinary Approach: ○ Collaboration between ophthalmologists, oncologists, and radiologists ensures comprehensive care.
